# National Campaign to Prevent Falls in Construction — United States, 2014

**Published:** 2014-04-25

In 2010, the 9.1 million U.S. construction workers (including self-employed workers) accounted for 7% of the national workforce (1). According to data from 2011, the rate of fatal injuries in construction was the second highest of any U.S. industry (2). Deaths and injuries from falls represent a critical, persistent, yet preventable public health problem. In fact, falls on construction sites are the leading cause of death in the industry (36% in private industry in 2012) (3). Many construction occupations require working in high places and climbing ladders or scaffolds on a daily basis. Many workers in construction trades are required to work from heights almost every day. Nearly 60% of workers in construction production occupations work at heights at least once a month, and many stand or climb on ladders or scaffolds during half of their work time (4). Safe construction requires skilled workers and responsible employers. The leading fatal events in construction are falls related to roofs, scaffolds, and ladders (1).

CDC’s National Institute for Occupational Safety and Health (NIOSH) has engaged construction sector stakeholders through a government-labor-management partnership representing state and federal government agencies, professional organizations, trade associations, labor organizations, and private industry to develop a national campaign aimed at construction contractors, onsite supervisors, and workers to address and reduce falls, fall-related injuries, and fall-related fatalities among construction workers. On Workers’ Memorial Day, April 28, the Occupational Safety and Health Administration and its stakeholders, including NIOSH, will formally announce its sponsorship of a National Safety Stand-Down to Prevent Falls in Construction during June 2–6. Additional information is available at http://www.osha.gov/stopfallsstanddown.

The Stand-Down is a voluntary event for construction-related employers to speak directly to employees about fall hazards and to reinforce the importance of fall prevention requirements. Modeled on U.S. military programs, the Stand-Down is a part of the national information and media construction falls prevention campaign developed through this partnership. Program sponsors encourage broad engagement and promotion across the United States, including by state agencies and public health practitioners.
